# Surveillance of acute flaccid paralysis in Akwa Ibom State, Nigeria 2004–2009

**DOI:** 10.4314/pamj.v9i1.71208

**Published:** 2011-07-22

**Authors:** Bassey Enya Bassey, Alex Gasasira, Pamela Mitula, Umoh Utobong Frankson, Johnson Adekunle Adeniji

**Affiliations:** 1 World Health Organization, UN HOUSE, Plot 617/618, Central Area District Garki, FCT, Abuja, Nigeria; 2 Epidemiology Unit, Ministry of Health, Uyo, Akwa Ibom State, Nigeria; 3 WHO National Polio Laboratory, Department of Virology, College Of Medicine, University Of Ibadan, Ibadan, Nigeria

**Keywords:** Acute flaccid paralysis, Surveillance, Poliomyelitis, Nigeria

## Abstract

**Introduction:**

The last case of wild polio virus transmission occurred in Akwa Ibom state in October 2001; however, combination high routine immunization coverage with OPV, high quality AFP surveillance, mass immunization campaign in which two doses of potent oral polio vaccine is administered to eligible children and mop-up campaigns in areas with identified immunization or surveillance gaps has help the state in maintaining a free polio status for over ten years. This study was carried out to describe the characteristics of reported acute flaccid paralysis cases between 2004 and 2009, and to evaluate the performance of the acute flaccid paralysis surveillance system using indicators recommended by the World Health Organization.

**Methods:**

A retrospective study was conducted among children, 0-15 years, by the World Health Organization (WHO) and Epidemiology unit of State Ministry of Health (SMOH), Uyo. The demographic characteristics and the results of isolation and identification of polio and other enteroviruses in stool samples sent to the WHO Polio Laboratory Ibadan for cases was analyzed.

**Results:**

A total of 521 cases of AFP (270 males and 251 females) aged 0 month to=15 years were reported by the surveillance system between 2004 and 2009. Those below 5 years of age accounted for 82.5% of cases reported and investigated. Of the 521 cases investigated 512 (98.3%) received at least three doses of oral polio vaccine, while 9(1.7) never received any oral polio vaccine (zero-dose). In all 5.1% of the isolates were Sabin, 7.9% non polio enterovirus (NPEV) and 2.3% were classified by national expert committee as compatible with poliomyelitis. There was consistent and steady increase in three critical indicators; Non polio AFP rate in children <15 years from 4.5 to 6.4 per 100 000 population, proportion of AFP cases with 2 stool specimens collected within 14 days of onset of paralysis from 57% in 2005 to 91% in 2009 and proportion of Local Government Areas (Districts) meeting both core indicators from 23% in 2005 to 87% in 2009. The highest numbers of cases were seen in the months of March, May and September.

**Conclusion:**

This study showed high levels of surveillance performance with some challenges in reverse the cold chain system, the continuation and sustained AFP case detection, prompt investigation and response, improvement in the reserve cold chain system would achieve optimal standards recommended by WHO and might provide a good model for the eradication of poliomyelitis.

## Introduction

Poliomyelitis is a highly infectious disease caused by the Poliovirus in the genus Enterovirus. It affects mainly children under 5 years of age and causes paralysis in 1:200–1000 infections [1]. In 1988, the World Health Assembly of the World Health Organization (WHO) resolved to eradicate poliomyelitis by 2000 [2]. In 1996 Nigeria started acute flaccid paralysis surveillance and in 2000 the Ministry of Health adopted a WHO-recommended virological classification scheme to replace the clinical classification scheme in use since 1991 [3]. With the new virological criteria, final confirmation of Poliomyelitis depends on the isolation of wild poliovirus in the laboratory [4].

Acute Flaccid Paralysis (AFP) is defined as acute or sudden onset of weakness or paralysis of a limb characterized as flaccid (reduced tone) in a child<15 years of age [4]. The surveillance of acute flaccid paralysis is the detection, investigation of flaccid paralysis of new onset in children under 15 years or any other suspected poliomyelitis case in a person of any age.

In Akwa Ibom State Polio eradication efforts have been highly successful, with the last case of wild poliovirus reported in October 2001. Since then, acute flaccid paralysis (AFP) surveillance has reached high levels of performance, with AFP rates of more than 6.4 per 100 000 children under 15 years of age being detected. Only AFP cases with wild poliovirus in stool specimens are confirmed as polio, while those with adequate stool specimens that are negative for wild poliovirus are considered as non-polio cases. Adequate stool specimens are defined as two stool specimens (8–10 g) collected within 2 weeks after onset of illness, with an interval of more than 24 hours between collections, transportation on ice, and arrival at the laboratory in good condition (no desiccation, no leakage)^5^.

The WHO developed a set of performance indicators to ensure that that AFP surveillance is properly conducted, maintained and operated uniformly., This paper discusses the AFP surveillance findings from 2004 to 2009, evaluates the system and identifies components that requires improvement.

## Methods

In total, there are 324 AFP reporting sites in the 31 LGAs (districts) covering the entire state, with one hundred and eighty four AFP focal persons responsible for AFP surveillance at the health facility and community level, while thirty-one disease surveillance and notification officers are at the LGA (district) level, four AFP focal persons and one WHO surveillance officer at the state level.

Every case of AFP in those aged 0-15 years has to be notified to LGA DSNO, SMOH and WHO by the AFP focal persons through form AFP F001: Immediate AFP case notification form, to the LGA (District) DSNO and AFP C101: Case Investigation form for acute flaccid paralysis to the SMOH and WHO with clinical and epidemiological information.

Clinical samples for virological investigation consisting of two stool specimens taken 24–48 hours apart within 14 days of onset of paralysis were collected for each case and transported under cold condition in giostyle containing frozen ice packs to the National Polio Laboratory Ibadan. Laboratory analysis was done by isolating the virus in RD and L20B cell lines followed by intratypic differentiation using the conventional/ real time PCR method.

All tests were carried out according to the standard protocols recommended by WHO and cases classified based on clinical and laboratory information, poliomyelitis: Poliovirus identified in the stool specimen; non polio: cases without poliovirus in stool specimens and no residual paralysis at 60 days; polio compatible: cases not definitely classified as non-polio on the available information. Acute disease with residual paralysis after 60 days or death or loss of the subject during follow-up, in which it was not possible to collect at least 2 stool samples within 14 days from the onset of paralytic symptoms; vaccine-associated paralytic poliomyelitis (VAPP): paralytic disease caused by vaccination with OPV. The association is thought to be valid if the vaccine is administered on the same subject in a period of time from 4 to 30 days before the onset of symptoms.

## Results

A total of 521 cases of AFP (270 male and 251 female) aged 0 month to = 15 years were reported by the surveillance system between 2004 and 2009. Those below 5 years of age accounted for the largest proportion (82.5%) of cases reported and investigated (Table 1).

**Table 1:** Age and Sex distribution of AFP cases reported to the surveillance system, 2004–2009

Variables		N	%
**Age ranges**			
	>50 years	25	67.6
	30–50 years	10	27
	<30 years	2	5.4
**Educational**			
	Never attended school	18	48.6
	Primary school	12	25.5
	Secondary school	7	18.9
**Residence of participants**			
	Within Salima District	32	86.4
	Neighbour district	3	8.1
	From Mozambique	1	2.7
	Not documented	1	2.7
**Profession of participants**			
	Unemployed	30	81
	Small business	2	5.4
	Civil servant	2	5.4
	Not documented	2	5.4
	Brewer	1	2.7
**Parity**			
	6–10 pregnancies	20	54
	4–6 pregnancies	11	29.7
	1–3 pregnancies	4	10.8
	>10 pregnancies	4	10.8
**Total**			**37**

Table 2 illustrates the number and vaccination status of acute flaccid paralysis cases reported to the surveillance system in six years. In 2005, the number of AFP cases reported was 58 (11.1%), owing to improved AFP surveillance performance in the state there was remarkable increase in the number of cases reported to 70 in 2006, 84 in 2007, 104 in 2008 and 133 (25.6%) in 2009. One indicator of the effectiveness of immunization activities is the proportion of children with non-polio acute flaccid paralysis (AFP) who never have received oral poliovirus vaccine (OPV). In total, this proportion declined from 3.4% in 2005 to 1.5% in 2009.

**Table 2:** Characteristics of prescribed medicines in an audit of deaths from cervical cancer in a Palliative Care centre, Central-East Malawi

		N	%
**Number of drugs prescribed (n: 37)**			
No drug prescribed		4	10.8
One drug		1	2.7
Two drug prescribed		5	13.5
Three drugs prescribed		4	10.8
Four drugs associated		10	27
Five drugs		5	13.5
More than 5 drugs		6	16.2
			
**Prescriber of drugs**			
Clinical officer		14	37.8
Senior registered nurse		15	55.5
Registered nurse		5	13.5
Not sure		3	8.1
			
**Types of prescribed drugs(37)**			
			
**Opioids**		19	51.3
	Codeine	10	27
	Liquid morphine	9	24.3
			
**NSAID**^a^		**34**	**91.8**
	Ibuprofen	22	59.4
	Indomethacin	1	2.7
	Diclofenac	5	13.5
	Paracetamol	1	2.7
	Acid acetyl salicylic	1	2.7
			
**Antibiotics**		**33**	**89.1**
	Metronidazole	23	62.1
	Cotrimoxazole	5	13.5
	Nystatin	1	2.7
	Erythromycin	1	2.7
	Amoxicillin	2	5.4
	Ciprofloxacin	1	2.7
			
**Laxatives**		**3**	**8.1**
	Bisacodyl	1	2.7
	Pawpaw leaves	1	2.7
	Pawpaw seeds	1	2.7
			
**Steroids**		**9**	**24.3**
	Kenacort	8	21.6
	Dexamethazone	1	2.7
			
**Anti-anaemic**			
	Iron	10	27
			
**Antiemetic**	Metochlopramide	12	32.4
			
**Others**^b^	12		32.4

a Non steroidien anti-inflammatory drugs

b Others are haloperidol, clorazepate, magnesium trisilicate, multivitamins, diuretics, amitryptilline, Expectorants and disinfectant

Forty one enteroviruses were isolated from the 1042 stool specimens investigated between January 2004 and December 2009, while twenty seven were vaccine-related Sabin strains. The National Expert committee classified 12 (2.3%) cases as Polio compatible according to the virological flowchart (Table 3).

**Table 3:** Symptoms prevalence among patients in an audit of deaths from cervical cancer in a Palliative Care centre, Central-East Malawi

	N (37)	%
Pains	15	55.5
Haemorrhage	13	35.1
Watery discharge	7	18.9
Urinary symptoms	4	10.8
Weakness	4	10.8
Sleeping problems	3	8.1
Abdominal distension/fullness	2	5.4
Oedema	2	5.4
Breathlessness	1	2.7
Vomiting	1	2.7
Loose of stools	1	2.7
Anorexia	1	2.7

The WHO defined the target of at least 2 AFP cases per 100 000 in children under 15 years as an indicator for measuring the sensitivity of AFP surveillance. This target was consistently achieved from 4.5 in 2004 to 6.4 per 100 000 population in 2009. The proportion of AFP cases with 2 stool specimen collected within 14 days of onset of paralysis remain consistently above the target of = 80%, except in 2005 where it declined to 57%, while the proportion of LGAs (districts) meeting two core indicators (Non-Polio AFP rate and Stool adequacy) rose steadily from 23% in 2005 to 87% in 2009 (Table 4).

**Table 4:** Performance indicators of AFP cases reported to the surveillance system, 2004–2009

Indicators	Target	2004	2005	2006	2007	2008	2009
							
Non polio AFP rate in children <15 years	≥2	4.5	3.3	3.9	5.1	5.0	6.4
Proportion of AFP cases with 2 stool specimens collected within 14 days of onset of paralysis	80%	97%	57%	87%	92%	95%	91%
Proportion of district making monthly reports including zero reports	80%	100%	86%	100%	100%	100%	100%
Proportion of AFP cases investigated within 2 days of notification	80%	100%	97%	100%	100%	100%	100%
Proportion of stool specimens arriving at national level within 3 days being sent	80%	100%	100%	100%	100%	100%	95%
Proportion of stool specimens arriving at the lab in good condition	90%	100%	100%	100%	99%	100%	100%
Proportion of stool specimens from which non polio enterovirus was isolated	10%	2.8%	3.4%	9.0%	12%	8%	5%
Proportion of stool specimens for which lab results were sent within 28 days of receipt at the lab	80%	100%	100%	100%	100%	100%	100%
Proportion of LGA(District) Meeting both core indicators	80%	100%	23%	58%	74%	81%	87%

## Discussion

The primary objective of acute flaccid paralysis surveillance is to detect, investigate, report, disseminate and promptly implement control measures. No wild polio virus has been reported in Akwa Ibom state since October 2001. However, the importation or reintroduction of Poliovirus from endemic states remains a threat, this underscore the importance to continue and sustain surveillance for AFP in children younger than 15 years until global eradication and certification is achieved.

We evaluated the age distribution of AFP cases which is also a risk factor. Four age categories were incorporated in the study, aged zero to one year, two to five years, six to ten years and eleven to those less than 15 years. In this study, the proportion of AFP cases 82.5% recorded in children aged 5 years and below was lower than the 90% that reported in India [6]. Similarly, another study in Ibadan, southwestern Nigeria also reported a lower prevalence (74.3%) among this age group of children [7], while a much lower prevalence of 37% was also reported in Marches region, Italy [8],

The isolation of Non Polio Enterovirus (NPEV) is an indicator used to evaluate the integrity and viability of stool specimen dispatched to the laboratory for viral isolation. It is expected that at least 10% of stool specimens dispatched to the laboratory should yield NPEV. Findings in this study shows that average enteroviruses isolation is 7.6% this finding is close to the findings reported in another study conducted in Bahawalpur, Pakistan where NPEV were isolated from 8.5% of AFP cases (9). The frequency of NPEV isolation in our study was however lower than the 34% reported in India [10]. Another study also in India had isolated NPEV from 20% of acute poliomyelitis cases [11], while 17.6% was also reported in Egypt [12] and 14.6% in Nigeria [13]. The variations observed may be attributed to factors such as differences in the specificity and sensitivity of laboratory methods and test kits, stool specimen collection, handling and transportation. The low NPEV isolation in this study may be attributed to poor stool collection, poor reverse cold chain and failure to adhere to standard procedures in the collection, handling and shipment of stool specimens. The implication of this low NPEV rate is that circulating wild polio viruses may be missed by the surveillance system.

In total, twenty-seven (4.7%) of the 521 acute flaccid paralysis cases yielded vaccine related Sabin strains. Isolation of these strains indicates vaccine-associated paralytic polio, a rare adverse event following ingestion of live oral polio vaccine. The mechanism of vaccine-association paralytic polio is believed to be reversion of the virus from its attenuated form to the virulent form [10]. Reversion is believed to occur in almost all vaccine recipients, but rarely results in paralytic diseases, which is more likely seen in immunodeficient children [10].

The 3% of compatible poliomyelitis cases reported during 2004–2009 offered a unique opportunity to document this subgroup of AFP cases, which is becoming more important as eradication progresses. Understanding the patterns of occurrence of compatible cases can help to pinpoint weaknesses in the AFP surveillance system and indicate what corrective measures should be taken [14]. All the 12 compatible cases were assessed 14 days after the onset of paralysis and found to be affected by residual weakness. These cases were classified as compatible because of inadequate stool collection, the main factor in which was a long interval between the onset of paralysis and case notification. Additional factors contributing to delayed stool collection should be identified and addressed so that this component of the surveillance system can be improved. In order to reduce the number of compatible cases it is necessary to minimize the time between the onset of paralysis, case notification and investigation. Factors such as weak links at this level may include delays in case presentation to the reporting network, delays between notification and the investigation of reported cases [14]. The second most common factor leading to the classification of AFP cases as compatible with poliomyelitis was the death of patients for whom two adequate stool specimens had not been obtained [14].

The present study confirms the improvement in AFP surveillance in a poliomyelitis low-risk area of southern Nigeria. According to state data for 2004–2009 from the same sources, the average rates of non-poliomyelitis AFP rose from 4.5 to 6.5 per 100 000 children, with all cases undergoing laboratory diagnosis. This reporting rate was slightly lower than to the national the 7.6 to 7.1 per 100 000 children<15 years) [15]. Five other states achieved AFP surveillance performance indicator rate in 2009 as follows: Bayelsa 8.67, Cross River 5.13, Delta 7.43, Edo 8.35 and Rivers 3.85 [15]. The present results demonstrate that a significant proportion of AFP cases are reported in the low-risk areas in 2004–2009. Subsequently, it was confirmed that reporting and laboratory diagnosis improved markedly, reaching approximately 80% for both indicators. Since active surveillance was conducted in all prefectures of the thirty–one districts, the observed changes were not independent of the recent improvement in AFP surveillance throughout the districts. Another factor that strongly influences the quality of AFP surveillance is adequacy of stool specimens, the frequency of adequate stool specimens ostensibly exceeded 80% district-wide. The WHO AFP surveillance performance indicator is 1 Non-Polio AFP case per 100,000 children less than 15 years of age, which is an international standard to assess the sensitivity of a national AFP surveillance programme. Non-Polio AFP rate has a directly relationship with population, increase in population figure results in high NPAFP rate expected to be recorded to meet the minimum target of NPAFP=1 per 100,000 population.

Although active surveillance detected a number of cases of clinical poliomyelitis, it was also apparent that as AFP surveillance developed many cases were reported late and then subjected to classification by the expert committee. Of the 512 cases of AFP cases in 2004–2009, only 12 were finally considered to be genuine compatible cases with poliomyelitis, but there was no geographical clustering and it is hard to imagine that these cases could be associated with wild poliovirus. In the state, annual campaigns of supplemental immunization: national immunization days (NIDs) and sub-national immunization days (SNIDs) continued nationwide and no outbreak of indigenous wild poliomyelitis was detected anywhere in the state after 2001, despite the existence of high-level virus surveillance [16]. Although active surveillance and analysis of clinical poliomyelitis cases were shown to be useful, the results clearly indicate that case reporting by AFP surveillance and subsequent laboratory diagnosis were essential to evaluate the interruption of wild poliovirus circulation.

Vaccine coverage among AFP cases indicates that coverage levels with 3 doses of OPV were uniformly high ranging from 96.6% to 100%. This high vaccination coverage perhaps left no room for susceptible children who may help to sustain transmission of poliovirus in the community. It is important to note that lowered immunization coverage may also have serious consequences in countries that use OPV, as was recently demonstrated by outbreaks of poliomyelitis due to circulating vaccine derived polioviruses (cVDPV) in Nigeria [17], Hispaniola, (Dominican Republic and Haiti) [18], the Philippines [18] and Madagascar [19]. Consequently, high coverage of polio vaccination is not only important in the period until the eradication of the wild type polioviruses from human circulation, but for as long as live OPV is in use.

## Conclusion

In general, the surveillance activity is satisfactory even if in presence of some criticalities in biological samples collection. The continuation of surveillance, in addition to the maintenance of current levels of performance, will tend to a further and more detailed sensitization of clinicians and other health workers involved, in order to obtain spontaneous and prompt reporting, and to achieve the optimal standards recommended by the WHO both in the collection of stool specimens samples and the availability of 60 days follow-up on cases with inadequate stool, Sabin, lost to follow-up, with the goal of eradicating polio from country. The state performance indicators are good because the Disease Surveillance and Notification Officers (DSNOs) who work at the LGA (district) level are well motivated through monthly stipend of $35 and $134 from WHO and the state Government respectively, the WHO state office and state ministry of health (MOH) also undertake data collection, collation and analysis, this form the basis with which feedback is provided to the LGA DSNOs and the health facilities. WHO funds monthly review meetings through which feedback is provided and intervention planned, this also provides a platform to share best practices. There is also close supportive supervision visits to the LGAs and Health Facilities from the state WHO office and officers from the MOH, while best practices from states are shared during zonal and country review meetings, the WHO office also publish best practices monthly.
